# Dr. Kinglake, on the Cure of Arthritis

**Published:** 1801-11

**Authors:** Robert Kinglake

**Affiliations:** Chilton super Poldon


### *To the Editors of the Medical and Phyſical Journal.*

Gentlemen,

Arthritic affections have long been denominated the *opprobria medicorum*; nor have medical practitioners diſcovered much ſolicitude to acquit the healing art of ſuch a degrading charge of incompetency. In no inſtance of diſeaſe has prejudice more arbitrarily uſurped the empire of reaſon than in the treatment of gout. The crude notion ſuggeſted by the humoural pathology of the ancients, that every diſtemper characteriſed by cuticular determination ſhould be conſidered as originating from ſome morbid condition of the fluids, requiring ſpecific expulſion from the ſyſtem, led alſo to the concluſion that gout was to be ſimilarly rejected; hence the routine practice of foſtering arthritic inflammation by the topical uſe of increaſed temperature, and the internal employ of ſtimulant medicines, with a view to obviate its retroceſſion, and to enſure its final extinction on the part affected. This procedure has invariably appeared to me to be repugnant to the indication of relief furniſhed by every conſtitutional and local feature of the diſeaſe. Obſervation and reflection have forced on my conviction conviction the *fact*, that however loofe the analogy might be between the refpective proximate caufes of ordinary phlegmonous and arthritic inflammations, the refemblance is fufficiently clofe in the degree of concomitant temperature. In *both*, the vafcular actions of the fyftem, and of the part affected, generate a morbid excefs of heat, alike referable to diftempered conditions of motive power. Impreffed then with the perfuafion that with regard both to inordinate temperature, and to its general as well as topical manifeftation, a radical fimilitude fubfifts between thefe *nominally* different inflammations, it appeared to me ftrictly warrantable to inftitute a perfectly fimilar plan of cure; the event has fully verified its probable utility, as will be evinced in the detail of the fubfequent cafes.

A young man about twenty-five years of age, of an healthy temperament, and without any known hereditary predifpofition to arthritic affection, had been, during three or four preceding years, attacked at leaft once, fometimes twice, in the courfe of twelve months, with a fevere fit of the gout; it ufually affected the great toe of one foot only, occafionally alfo both knees, and one or more of the finger joints. On thefe feveral parts were permanent inflammation and tumefaction, arthritically characterifed by the ufual fhining afpect, and exquifite fenfibility to either touch or motion; morbidly affociated irritation alfo arofe in different parts of the fyftem, but did not become fo concentrated as to be either durable or ferioufly violent.

The medical advice given the patient on thefe occafions by different practitioners, was to confider the complaint as a violent degree of gout, to cover the affected parts with flannel, to fuftain the powers of life by the occafional ufe of ftimulants, and refignedly to await the ordinary folution of the difeafe. This not lefs hackneyed than pernicious admonition had been repeatedly complied with, at the expence of feveral months painful durance, and the confequent lofs of much conftitutional ftrength. In the attack preceding the laft, after feveral weeks confinement, and the injurious effects of high temperature, my advice was defired; the ligaments of the great toe joints, of one foot, of both knees, and the wrift of one hand, were at that time much inflamed, thickened, and fwollen, extremely painful on the flighteft preffure, or effort at motion; morbid fympathetic irritation had alfo pervaded the fyftem, and been more or lefs permanently arrefted and evolved in relative fymptoms, on the brain, ftomach, and bowels; conftitutional atony had likewife advanced to an alarming height, and diftempered irritability prevailed in a threatening degree.

Under thefe feveral unfavourable circumftances, no time was to be loft; much mifchief had already been incurred by awaiting a natural termination of the difeafe.

The local inflammation and irritation appearing to be the chief fources of the evil, it feemed highly expedient to fubdue what in my eftimation conftitutes the efficient caufe of thefe active fymptoms, *namely*, *excessive temperature*; this can only be indirectly accomplifhed by reduced heat, and no vehicle is fo fuitable to effectuate it as cold water.

In oppofition then to every prejudice, equal parts of cold water and acetated water of ammonia, (aqua ammoniæ acetata*) were directed to be conftantly applied to the affected parts by means of cloths wetted in that fluid, renewing them every half-hour, or even at fhorter intervals if any fenfe of morbid heat fooner returned. With this external remedy was combined the internal exhibition of camphorated tincture of opium (tinctura opii camphorata) and the ammoniated tincture of guaiacum (tinctura guaiaci ammoniata) in dofes of two drachms each, repeated every eight hours. The relief obtained from the topical application of cold to the parts affected was immediate. The efflorefcence, fwelling, and ftiffnefs were fo far diminifhed within twelve hours, as to admit of moderate preffure, and even voluntary motion of the affected joints with but little inconvenience. The co-operative influence of the internal medicine feemed to calm the agitated ftate of the fyftem, and to difpofe to fleep; but it manifeftly, at the commencement, had no fenfible effect on the local irritation, for, on the third and fourth days, if (as fometimes happened) the application of the cold mixture was not duly purfued, though the medicine was regularly exhibited, a painful fenfe of heat and renovated tenfion promptly recurred.

After five days profecution of this treatment, four diftinct fources of local irritation were annulled; the fyftem became recruited, and pervaded by an equal diftribution of improving vital energy; the appetite returned, digeftion amended, and the nights were no longer fleeplefs. This convalefcent procedure happily terminated in the courfe of one month in the full reinftatement of health.

After an interval of nine months perfect freedom from every fymptom of arthritic affection, this patient fuffered a renewed attack;

* My motive for conjoining the acetated water of ammonia (aqua ammoniæ acetata) with common water, was not with a view to any difcutient quality which might be fuppofed to refult from the mixture, but merely to avoid exciting any dread in the Apothecary who furnifhed it, againft the employ of cold water *alone*, which would probably have proved an impediment to its due application.

attack; the ſame parts were preponderately affected with gouty irritation, as in the former inſtance, but with additional violence. It had prevailed upwards of a fortnight before my advice was taken, during which period the ordinary medicinal treatment in theſe caſes (and which has been already mentioned) was employed, and with the ſame unavailing effect and deleterious conſequence. The diſorder became hourly exaſperated; every ſymptom, particularly thoſe of exceſſive temperature and exquiſite ſenſibility, had been much exacerbated, and ligamentous thickening and inaction had induced abſolute inability to move the affected joints without aſſiſtance.

The experience of the paſt had been too unequivocally demonſtrative of the beneficial influence ariſing from the topical application of reduced temperature, not to be again reſorted to. The plan of treatment before recited, was renewed, and with preciſely ſimilar effects. In the courſe of three days, the topical employ of the cold fluid had ſo diminiſhed the local ſwelling and irritation, as to unfetter the confined joints, and to reſtore the power of tolerable loco-motion. The medicine before employed was alſo reſumed, with the evident advantage of calming the irregular action of the ſyſtem by equalizing the circulation of the vaſcular fluids, and thereby reinſtating the cuticular and every other diſturbed ſecretion. In the courſe of three weeks, convaleſcence was ſo far advanced, as to leave nothing more to complete the cure, than the reſtoration of natural tone to the debilitated conſtitution. At this time, now upwards of two months from the date of the attack, this final benefit has been fully accompliſhed by the conjoint aid of bark, exerciſe, and a well-regulated courſe of nutritive diet.

The moſt ſceptical muſt admit, that the highly ſalutary effects of diminiſhed temperature, in this caſe, are beyond all controverſy. In both inſtances, the neglect of it had permitted the diſeaſe to go to an unpromiſing length; neither natural effort, nor ſuperadded aſſiſtance, ſeemed competent to effect the change neceſſary to ſubdue the inflammatory and decompoſing proceſs of arthritic irritation. Morbid afflux of fluids on the affected ligaments, had cauſed the generation of new veſſels, which were, in turn, engaged in carrying on diſeaſed ſecretion and accretion. Reduced temperature abſtracted this diſtempered action; and by thus depriving the diſordered parts of nutritive ſupply, expoſed them to be ſpontaneouſly decompoſed, and borne away by exhalant impulſe and lymphatic tranſmiſſion.*

The

* The phraſe *lymphatic transmission*, is here ſubſtituted for the ordinary term *absorption*, it being my opinion that the lymphatic veſſels do not in any inſtance

The value of the evidence afforded by this cafe, is alfo much enhanced by its embracing two inftances, in which the curative influence of reduced heat in arthritic malady was equally ftrongly marked. Had the iffue, however, been fomewhat different, it might have been owing to that perpetual and infcrutable diverfity in temperamental fufceptibility, which perplexes and defeats every endeavour at difcovering any thing like uniformity in the effect of agents on the animal œconomy; but the exactnefs of the co-incidence appears to warrant the important conclufion, that reduced temperature is capable of fuch an abfolute degree of efficiency, as cannot be either refifted or modified by *slight* organic diffimilarity.

The fecond fubject of topical refrigeration under my direction in arthritic affection, was that of a woman aged forty, of an healthy temperament, who had been grievoufly tortured upwards of four months with gouty irritation inveterately ftationed on the articular ligaments of both ankles, one knee, and both wrifts; neither of the toes was affected, nor had the patient prior to this time experienced any fimilar complaint, yet the peculiarly gloffy tumefaction, efflorefcence, and exquifite fenfibility of the affected parts were eminently characteriftic of arthritic difeafe. Subordinate degrees of irritation occafionally wandered over the fyftem, with the feeming effect of affording temporary alleviation to the more fixed pain, but without exonerating the difeafed ligaments of their cumbrous inflammatory load; indeed, the relief appeared to confift rather in fomewhat diverting the attention from the accuftomed fource of grievance, than in its real diminution.

This patient had unfuccefsfully tried the probable modes of affiftance which reputable medical advice, during feveral months, could afford; at length, wearied and difcouraged by the fruitlefs effect of every endeavour, and having been informed of the benefit which had been rendered under my direction to the patient whofe two cafes have been juft ftated, my opinion was defired on her fituation. It appeared to me, at firft fight, to be fo clearly a cafe of morbid temperature habitually fixed by long ftanding, and bearing fo clofe an analogy to the preceding cafes, that a fimilar treatment was inftituted for her relief, with particular attention to the improvement of her digeftion, which indeed had been fo much impaired as to have greatly curtailed the due alimentary refources of vital fuftenance.

The topical application of cloths wetted in the cold mixture before

---

inftance *actively absorb*, but *passively admit* the redundant, exhalant, interftitial, or extravafated fluids forced into their *permanently open orifices* by arterial and mufcular impulfe.

before mentioned, afforded ſpeedy benefit, and its frequent renewal ſoon reſtored motive power, to the ſtiffened and diſabled joints. With the ſubſidence of the ſwellings, ceaſed alſo the morbid irritation that had reduced the natural energy of the ſyſtem to a wretched ſtate of atony; hence the powers of life were no longer ſquandered in diſeaſed motions, but recruited by the renewal of healthy action. Three weeks procedure in this mode of reſiſting local irritation by topically allaying redundant heat, had effected unexpected changes; at the end of one month a meſſage from the patient deſired that ſomething ſtrengthening might be directed, obſerving that the ſwellings and pain were wholly removed; that the diſabled joints had regained the power of voluntary motion, and that a retrieval of ſtrength ſeemed to be alone wanting to complete the cure; ſince that time, now upwards of two months, no intelligence has reached me concerning the patient, which induces me to preſume on at leaſt a *ſatisfactory*, if not a *perfect* recovery.

This caſe affords an inſtructive inſtance, that whatever may be the degree of prevailing debility, the exiſtence of morbid exceſs of temperance, whether local in the character of inflammatory affection, or general in the ſhape of febrile irritation, might be ſafely combated with rapid and perſevering reduction. The debility here preſent was inceſſantly advancing by the inordinate ſtimulant effect of the attendant irritation, and would have ſoon gone to the extent of fatal exhauſtion, had it not been obviated by the appeaſing and regenerating influence of diminiſhed heat.

The fourth caſe of this deſcription which you are requeſted to indulge me in citing, is that of a young man much addicted to intemperate drinking, who had been formerly ſubject, at irregular intervals, to gouty affection on the great toe of one foot only. In the laſt attack my advice was taken on account of an effloreſcent diſcolouration diffuſed over the whole foot. This additional appearance to the uſual more confined aſpect of gout, had induced much alarm for the imaginary conſequences. The arthritic inflammation originated on the ſecond joint of the great toe, was productive of inſufferable pain, and conſequently of ſevere ſymptoms of ſyſtematic irritation.

My former experience of the ſalutary effects of reduced temperature emboldened me to confide the welfare of this patient to its ſalutary influence. He was ſupplied with a pint bottle of cold water, (coloured for the purpoſe of diſguiſe) in which he was directed to moiſten a folded cloth, and to envelope with it the whole foot, renewing it as ſoon as the ſenſe of imparted coldneſs was overcome by that of returning heat; this was on an average about once in half an hour. The relief obtained was was immediate; in a few hours the affected joint could be voluntarily moved; by the following day the cuticular rednefs over the foot affumed a flightly livid hue, the tumefaction alfo diminifhed, and in the courfe of one week the patient walked about with tolerable convenience. No medicine was directed, and abftinence only from immoderate indulgence in fpirituous and fermented liquors was enjoined. A tight bandage, commencing at the affected toe, and fpirally continued over the foot and ankle to the knee, reftored in a few weeks the accuftomed tone to the enfeebled extremity; thus the ftrict *podagral* character of arthritic affection was arrefted in its progrefs, and falutarily terminated with unprecedented rapidity by the aid of diminifhed heat.

This cafe prefents an example of unmingled gout fafely and expeditioufly fubdued by the fole external ufe of cold water. Not the fmalleft unfavourable fymptom arofe in the train of its removal; on the contrary, the fyftematic irritation which appeared to have been excited and fuftained folely by the local pain, ended with the ceffation of its caufe; and every relic of morbid feeling, whether from habitual or atonic influence, fpeedily yielded to the renovated conditions of perfect health.

My obfervation of the benefit refulting from the topical application of reduced heat in arthritic inflammation, has yet been limited to the four preceding cafes. Some, indeed, who have been veterans in the diforder, and who, from having been prejudiced with the popular notion that its periodical return and due local fupport were effentially neceffary to health, could not be prevailed on to apply cold water, have yet, on my recommendation, fo far relaxed from employing high temperature as to difrobe the affected parts of flannel, and expofe them to the ordinary heat of the atmofphere; the confequence of this partial compliance has been fuch relief as to induce a perfeverance in the too reftricted remedy.

From what has been related, it appears allowable to infer, that high temperature, whether the caufe or effect of the morbid conditions of vital power which proximately conftitute gout, is fafely and fpeedily controulable by the fimple application of cold water; that the prevailing opinion relative to the critical nature of that difeafe on the extremities is liable to much diftruft; that the local depofit is not, as commonly fuppofed, a particular preponderance and detention of the conftitutional diforder, but that it originates in the parts themfelves, and is thence diftributed by affociated influence over the fyftem; that the longer the local affection endures, the greater probability there will be of morbid fympathies being generated and eftablifhed blifhed on the vital organs, which may terminate in rapid and painful death.

If thefe inferences be admitted, the falutary effects of early and inceffant low temperature, recited in the preceding cafes, will be eafily explicable; and it will follow that the true indication of cure in arthritic inflammation will confift in transferring, by cold media, the redundant heat of the part affected.

It may be demanded, if this refrigerant mode of topically abftracting the ftimulus of heat is fo efficacious, would not a general antiphlogiftic plan, as it is termed, powerfully cooperate in fubduing the morbid ftate of the affected parts? My obfervation enables me to anfwer this queftion by affirming, that fuch collateral aid is not neceffary, without warranting me in faying it would be pernicious; but it is rational to expect that it would have hurtful tendency by adding to the prevailing fyftematic debility, and thereby incurring the infeparable danger attendant on extreme weaknefs, and irritability.

The falutary influence of topical cold on inflamed furfaces does not depend on debilitating motive power, but in removing that immoderate excitement which always deranges and threatens, by progreffive exhauftion, the ultimate extinction of life; thus the tonic and invigorating effect of affufion with cold water in febrile affections, and of immerfion in various chronic difeafes, may be explained.

The moft fummary mode, perhaps, of reftoring ftrength in highly enfeebled and irritable ftates of vital motion, is to abftract all unneceffary, and particularly *painful* excitement. The occafional exclufion of light, avoidance of found, undue heat, redundant aliment, inteftinal conftipation, and mental anxiety, will often be found conjointly to promote the regeneration of more fyftematic energy in default of that power, than the beft managed employ of medicinal ftimulants.

*Chilton fuper Poldon,* I am, &c.

*Oct.* 12, 1801. ROBERT KINGLAKE.

