# Errata

**Published:** 1801-12

**Authors:** 


					Page 459, '? 2?? for temperance, read temperature.
Page 4.5 j, ). 41, for falutary, rzad iolitary.
EN.P OF VOL. VI,

				

